# Dental Examining Boards in the United States and Preliminary Education

**Published:** 1896-02

**Authors:** G. Carleton Brown

**Affiliations:** Elizabeth, N. J.


### DENTAL EXAMINING BOARDS IN THE UNITED STATES AND PRELIMINARY EDUCATION.[1]

BY G. CARLETON BROWN, D.D.S., ELIZABETH, N. J.

MR. PRESIDENT AND MEMBERS OF THE CENTRAL DENTAL ASSOCIATION,—At the request of your Executive Committee, I, in a moment of weakness, agreed to read a paper to this society on "Examining Boards and Dental Education," or something of that kind. I had no sooner made this fatal error than our ubiquitous chairman of the Dinner Committee, Dr. Meeker, appeared upon the scene, and demanded the title of the paper. As I had not the slightest idea what I was going to say, I could not think of a title, so I told him to call it anything he pleased; then I had to wait until the programme appeared to find out what I was to write about. When I did find out, it struck me that the title gave me license to talk about almost anything, so that a general *pot-pourri* on dental education is the result. I trust you will excuse the crude form in which it is served.

A *résumé* of the question in regard to the influence of examining boards on dental education would necessarily become a history of the advance of modern dentistry, and in saying this and in claiming a large share of this advance for the examining boards, I do not mean to detract a particle from the great work that has been done by the colleges; to them, first and foremost, belongs the credit for the advanced standing of the profession of dentistry to-day. But, I greatly doubt whether the colleges would have attained to their present standing, or have demanded the high standard now required of graduates, if it had not been for the examining boards. There are several ways of looking at why and how this influence came into existence; but I think the most rational is that the colleges are not working entirely from a philanthropic stand-point, while the examining boards are. The colleges are pecuniarily in-

---

[1] Read before the Central Dental Association of Northern New Jersey, December 16, 1895.

terested in their work, and are, of course, striving to make all the money they can, and in some cases more than they have any business to make,—but, as Rudyard Kipling says, "that's another story,"—while the members of the examining boards are sacrificing much time, and in nearly all cases are spending their own money, to elevate the standard of their profession; and this, without the slightest chance of any pecuniary benefit to themselves.

These facts being accepted, with the further statement that in spite of the antagonistic attitude assumed by some of the colleges towards the boards, the latter have none but the kindliest feelings to the colleges, and when they make criticisms and suggest certain reforms it is not because they love the colleges less, but their profession more.

The elevating of the standard of dentistry demanded by the profession must be done, to a great extent, through the educational mediums, and as public institutions they are certainly open to criticism if they fail to properly equip their graduates.

Any one who has been an unprejudiced observer of the changes in the teachings of the schools and the character of the material turned out since the establishment of the State examinations must admit that the boards have had an influence on the colleges, and in many instances have been the means of procuring for the student a higher and better education; this being the case, is it not the duty of the boards to continue the work by pointing out the defects which still exist, and by suggesting reforms?

The only question that now arises is, Should these discussions be confined to the national associations representing the colleges and the boards, or should the matter be openly and freely discussed by the profession at large? I consider the latter the proper course, hence this paper.

A few years ago it seemed the fashion for a man, when he thought he could not make a success of anything else, to either enter the ministry or take up dentistry as a profession; if the latter were his choice, the mere fact that he had not the preliminary education that would allow him to receive and assimilate the scientific and theoretical teachings of the profession did not enter into the question any more than did the fact of whether he had the requisite amount of mechanical adaptability.

The colleges recognize this by theoretically requiring a preliminary education; I say theoretically, for what they actually require hardly deserves even the name of education.

In making these charges I do not say that some schools may not give a proper preliminary examination, but I do know that the examination, if it exists at all in certain schools, must be a perfect farce. In making this statement I know I shall be borne out by members of any examining board who require a written examination. I will give you a few illustrations to demonstrate the point. These illustrations are taken from the papers of recent graduates and are exactly as written, none being selected unless plainly written so that no mistakes could be attributed to defective penmanship.

*Q.* What relation to dental caries do the micro-organisms bear?

*A.* Dental caries is a destruction of the tooth substance which is hard and micro-organisms is effect the soft parts of a body.

*Q.* What acid is produced by the micro-organisms?

*A.* Toxic acid is produced by the micro-organisms.

*Q.* How?

*A.* By the action of them upon the soft tissues, these be open.

*Q.* What is plethora?

*A.* Is a disease of the Pleura.

*Q.* What is disease?

*A.* Is the disturbance of the equlebrum or preversion of circulation.

*Q.* What is fever?

*A.* the riseing of the tempeture caused by too much blood in a part.

*Q.* What is shock?

*A.* shock is the suddent checking of the nerves caused by accident.

*Q.* What is the difference between a narcotic and an hypnotic?

*A.* Narcotic acts on part the small intestines. Hypnotic acts on the whole large intestines.

*Q.* What are the stages of anæsthesia?

*A.* drowsiness sleepy feelings long breathing Stifness of muscles and relaxation of the parts.

*Q.* What are the effects of inhaling nitrite of amyl?

*A.* It produces krowness.

Now, as to a student's mechanical adaptability, granted a first-class education and the receptive qualities necessary for a good scientific education, of what avail is it "if he hasn't it in his fingers?"—as Dr. Eaton has so tersely expressed it,—he will never make a dentist, and of this fact the colleges have as yet taken no notice; they might perhaps refuse to graduate him at the end of his three years course, even if he stands first in his class in theory; but, would they do this? And if they did, would it not be pretty hard on the student? But, is it not worse to have an incompetent man turned loose upon the public? And here is where the examining boards come in again, as our friend, Professor Flagg, says, "the board steps up and says 'Hold!'" and the young man steps out into "innocuous desuetude" with three of the best years of his life wasted.

This is certainly hard on all concerned,—the boards, the college, and the student. But, hard as it may be, the boards must do their duty as State officials and servants of the public. There is no question about its being hard on the student; the college from which he graduated should feel a twinge of remorse for having taken his time and money and failed to give him what he has paid for; they (the colleges) may have done all in their power, but if the student did not "have it in his fingers" to start with, all the colleges in the country could not have given it to him; their mission being only to develop. Where is the remedy? Many, I am sure, must have given the subject thought, but the first practicable suggestion that I have seen comes from Dr. Crouse, who says, "A young man may be bright, a good student, and well grounded in the classics, and yet, an attempt to make a dentist of him would destroy his usefulness in life, make him a detriment to the community in which he practises, and not a credit to the dental profession. Therefore, the first six weeks of the college course should be spent in finding out who are properly qualified, by nature as well as by training, to be dentists; and those who are not fit should have their fees refunded and should be persuaded to select a more suitable calling in life. In short, the 'plucking' should be done at the beginning of the college course rather than at the end." Here is something for the faculties to think about; and by adopting some such system they will save the boards many unhappy moments.

And while they are occupied with this subject, it would be well to look more carefully after the training of the students in the practical departments. It is a noticeable fact that since the increase of the term of pupilage,—from two to three years,—while the scientific and theoretical knowledge of the graduates has increased to a marked degree, the practical has not only failed to keep pace with it, but has actually decreased. Are we, as a profession, sacrificing the practical to the theoretical? Whoever are leading in this matter, the profession or the professors, damage is being done and a halt should be called.

According to the reports given by students, the means and apparatus in some of the colleges for pursuing the practical studies are absurdly inadequate, in some instances there being only one blow-pipe for the use of several hundred students, within many cases no chance of obtaining instruction in its use even then; the demonstrator, so called, being engaged in the more important (?) work of looking after the business part of the insertion of artificial dentures, for a consideration, said consideration, by the way, netting a good round profit to the college. In fact, the colleges seem to be giving instruction only in the direction in which there is a direct pecuniary benefit to themselves, instead of educating the student to a higher standard of general proficiency in mechanics.

I have had graduates tell me that the case they soldered before the examining board was the first piece of metal work they had ever done, and one man acknowledged that he had never had a blow-pipe in his hand before. The colleges may say that this evidence is not trustworthy, as it comes from men who, after having made a poor piece of work for us, are trying to throw the responsibility on the college; this might have more weight if it were not for the fact that the story comes from so many different graduates, and is so amply corroborated by the work they do at the examinations. If you will carefully examine the specimens which I herewith submit, I think you will come to the conclusion that there are reforms needed in this direction. The accompanying specimens have been selected and mounted by Dr. Barlow,—who has charge of the mechanical department of the New Jersey board,—from work done within the last year, as part of the practical examination required from each applicant.

Have the men who did this work received a proper education to enable them to practise dentistry?

I think this will prove to your satisfaction that in some cases the practical side is being sacrificed to the theoretical. That others have noticed the same thing is shown by the following extract from the report of the Committee on Practice of the New York State Society, read at Albany, May 8, 1895. "Our institutions of learning should take heed that the manual skill of their graduates does not suffer on account of increased theoretical training."

The colleges may claim that every student has to submit a satisfactory piece of metal work as part of his examination and a prerequisite to graduation; true, but do they inquire carefully into the question of who made the piece? That, however, is part of the other story I am coming to later.

Leaving the mechanical department for the operative, we perhaps find an improvement. But does not the effort to swell the college treasury overbalance the educational features? Are there not many teeth that by proper treatment could be saved sacrificed to make way for other work that would pay better, thus doing injury to patients and depriving the student of an important means of education? How many students, when they graduate, are really competent to contend with the complicated cases of pulpless or abscessed teeth?

If any of you will stop and think of the many difficulties you have had to contend with and master for yourselves, since you commenced active practice, that could have been greatly simplified by proper instruction from a demonstrator during your collegiate course, you will better appreciate the point I am trying to emphasize.

Is it not just as important that there should be a competent corps of demonstrators as of lecturers? Now, as a rule, there is one head demonstrator whose duty it is to apportion the patients and handle the gold and cash, these occupations leaving him little time to assist and advise the students in their work, these latter functions being left to undergraduates, who are appointed assistant demonstrators. If the colleges would provide a sufficient number of first-class demonstrators to be constantly on hand during clinic hours in order to personally instruct students in the correct diagnosis and treatment of cases, the percentage of failures before the examining boards would be materially decreased. These remarks will apply equally well to either department of the practical education in our institutions.

While on the subject of college clinics I cannot resist again alluding to the matter of fees. The present system employed in many institutions of having fixed prices for operations, places them about on a par with the so-called "associations" which have done so much to lower the standard of dentistry in the public mind. In both cases the bulk of the work is done by inexperienced and incompetent men; in the associations, because that kind of help comes cheap, and in the colleges, because the workmen are students. These methods have taken away any right which the clinics might once have had to be called free.

One means of cementing the colleges and the profession more closely together would be for the former to return to the old method of simply charging for the material used, and making the clinic a dispensary in the true meaning of the word.

The method of procedure in the mechanical examination before the New Jersey board is as follows: Every candidate is required to make a metal plate, band it, grind and back the teeth, and invest ready for soldering before the board. Some time since, it became apparent that in a great many instances the character of the preparatory work and the final soldering differed so markedly that it was impossible that the same person should have done both. This led to an investigation, and it was found that there were persons who made a regular business of supplying students, who were to appear before the board, with plates ready for soldering; it was also found that these same persons were in the habit of making the graduating cases for students in the colleges, charging them different prices, according to style and finish. The board met this difficulty by requiring each applicant to make an affidavit stating that he did all the work on his plate himself, without assistance from any one.

The fact that a student can buy a plate, present it, and have it accepted as his own work in college, certainly confirms my previous statement as to the lax way in which the clinics are conducted. A demonstrator in the mechanical department certainly should know whether a graduating piece was made in the college laboratory or not. But, worse even than this comes the report that in some cases the demonstrators have themselves made these plates for the students, they, of course, being paid for the same.

Another reported condition of affairs in one of our colleges seems to me, if true, to deserve such unqualified disapproval from the profession that a continuance of such practice will henceforth be impossible. It is, in short, that a certain dental house holds such a large interest in one of our colleges that the students say they dare not buy their instruments from other houses, because by so doing they would jeopardize their chances of graduating. If this report is true, taken in connection with the other matters which I have laid before you, is it not time that the profession at large insisted on knowing more about the way in which our institutions are conducted; and, where reforms are needed, insist on their not only being introduced, but lived up to?

In conclusion, I wish to state that no charge in this paper has been aimed at any particular college, the defects mentioned being distributed among the different institutions; there are some however, that may, perhaps, lay claim to all. On the other hand, there are certain colleges to which these criticisms are in no way applicable.

Let the profession, the colleges, and the boards unite their forces and work together in this matter, and a higher standard is bound to result.

